# Midterm results after allogeneic simple limbal epithelial transplantation from deceased‐donor eyes in patients with persistent corneal epithelial defects due to limbal stem cell deficiency

**DOI:** 10.1111/aos.16760

**Published:** 2024-09-27

**Authors:** Jana C. Riedl, Joanna Wasielica‐Poslednik, Bert C. Giers, Francesco Buonfiglio, Norbert Pfeiffer, Aytan Musayeva, Adrian Gericke

**Affiliations:** ^1^ Department of Ophthalmology University Medical Center, Johannes Gutenberg University Mainz Mainz Germany; ^2^ Laboratory of Corneal Immunology, Transplantation and Regeneration, Schepens Eye Research Institute, Massachusetts Eye and Ear, Department of Ophthalmology Harvard Medical School Boston Massachusetts USA

**Keywords:** allogeneic, cornea, epithelium, limbus, transplantation

## Abstract

**Background:**

This study aims to characterize the clinical outcomes after allogeneic simple limbal epithelial transplantation (alloSLET) utilizing tissue from cadaveric donor eyes to address persistent corneal epithelial defects caused by limbal stem cell deficiency.

**Methods:**

We conducted a retrospective analysis of medical records from 20 patients, encompassing 24 eyes, who underwent alloSLET at least 2 years prior. The primary endpoint was the achievement of complete epithelialization of the corneal surface by corneal epithelium. Secondary endpoints included corrected distance visual acuity (CDVA) and postoperative adverse events.

**Results:**

The median postoperative follow‐up period was 36 months (range, 24–74 months). At 1, 3 and 6 months post‐surgery, 96% of eyes demonstrated epithelialized corneal surfaces, which declined to 71% at 12 months, to 54% at 24 and 36 months after surgery, and to 50% thereafter. There were no significant differences in graft survival between alloSLET performed alone versus in combination with penetrating keratoplasty. However, instances of graft failure were associated with postoperative elevated intraocular pressure (IOP) and a history of multiple amniotic membrane and corneal graft transplants.

**Conclusions:**

AlloSLET emerges as a viable mid‐term intervention for limbal stem cell deficiency‐associated non‐healing corneal epithelial defects in the absence of autologous limbal tissue. Our findings underscore the increased risk of graft failure in patients with elevated IOP and a background of multiple previous amniotic membrane and corneal graft procedures.

## 1 INTRODUCTION

Allogeneic simple limbal epithelial stem cell transplantation (alloSLET) represents a novel approach for addressing limbal stem cell deficiency (LSCD), a major clinical challenge in ophthalmology in cases where autologous tissue is unavailable (Haagdorens et al., 2016; Ruan et al., 2021; Yazdanpanah et al., 2019). Thus far, alloSLET has mostly been explored as a therapeutic avenue for LSCD subsequent to bilateral chemical injuries in both adults and children (Agarwal et al., 2020; Bhalekar et al., 2013; Iyer et al., 2017; Kunapuli & Fernandes, 2021; Mittal et al., 2016). Agarwal et al. (2020) conducted a comparative study evaluating the outcomes of alloSLET versus amniotic membrane grafting in patients with severe ocular chemical injuries, reporting a swifter epithelialization period and improved visual outcomes in eyes treated with alloSLET. Additionally, the efficacy of alloSLET in managing LSCD stemming from causes other than chemical injuries has been demonstrated (Riedl et al., 2021; Vasquez‐Perez & Nanavaty, 2018). A stable corneal epithelium was achieved in 71.4% of the eyes 1 year after alloSLET (Riedl et al., 2021). However, the major limitation of that study was the short follow‐up time. Of note, most previous studies utilizing other transplantation techniques of allogeneic limbal epithelium from cultured corneo‐scleral buttons, such as keratolimbal allografts (KLAL) and cultivated limbal epithelial transplantation (CLET), mostly reported on success rates of only 50% and below 2 years and longer after surgery (Miri et al., 2010; Pauklin et al., 2010; Shimazaki et al., 2004, 2007). The goal of the present study was to evaluate the outcome of alloSLET from cadaveric cultured donor corneoscleral buttons in patients with LSCD and non‐healing corneal epithelial defects with a minimum follow‐up period of 2 years post‐surgery.

## 2 METHODS

This is a retrospective, interventional case series, which was conducted at the Department of Ophthalmology, University Medical Center of the Johannes Gutenberg University Mainz. According to local law (‘Landeskrankenhausgesetz’ §36, §37), no ethical approval was required for this retrospective analysis.

### 2.1 Patients

Medical records of patients that had been treated for persistent corneal epithelial defects with allogeneic simple limbal epithelial transplantation (alloSLET) between September 2017 and September 2020 have been reviewed. The inclusion criteria were: (a) Patients who received alloSLET for a non‐healing epithelial defect with a history of failed epithelialization due to total LSCD mostly after at least one superficial keratectomy with amniotic membrane transplantation. Of note, in all patients no autologous tissue was available either because of bilateral involvement, because the patient was afraid to worsen the situation in the fellow eye and therefore refused biopsy or because the patient had only one eye. (b) A postoperative follow‐up time of at least 24 months. All patients had scheduled examinations at the Department of Ophthalmology of the University Medical Center of the Johannes Gutenberg University Mainz 1, 3, 6 and 12 months after surgery and yearly thereafter. The examinations included assessment of corrected distance visual acuity (CDVA), slit lamp examination and fluorescein staining. Patients were also regularly visited by their local ophthalmologist and referred to our clinic in case of any questions or abnormalities. Notably, of the 24 eyes presented in this study, the outcome of 14 of them has been reported 1 year after surgery in a previous study by us (Riedl et al., 2021).

The primary outcome measure was clinically defined as complete epithelialization of the corneal surface by corneal epithelium. Failure was defined by the presence of persistent epithelial defects, corneal melting, microbial keratitis or the need for repeat surgery. Additionally, progressive vascularization and conjunctivalization into the central 5 mm zone of the cornea were considered criteria for failure.

Secondary outcome measures were CDVA and postoperative side effects. Moreover, recipients' characteristics (age, the number of previous amniotic and corneal grafts, other eye diseases) and donor/graft and harvesting/culturing characteristics (donor age, endothelial cell count, death‐to‐harvest time, time of cultivation) were evaluated to determine risk factors for graft failure.

### 2.2 Surgery

Surgeries were performed by two experienced surgeons (A.G. and J.W.). The procedure has already been described in detail previously [9]. First, the fibrovascular pannus was removed by using a hockey knife and eye scissors. If necessary, bleeding was reduced by gentle cauterization. In patients with deep corneal ulcers or stromal scars, penetrating keratoplasty (PK) was performed after removal of the pannus. After the penetrating corneal graft had been sewn in, the epithelium was removed by using a hockey knife and a tying forceps. Subsequently, a cryopreserved amniotic membrane (Eye bank of Rhineland‐Palatinate, Mainz, Germany) was placed with the stromal side down on the cornea and bare sclera, attached with fibrin glue (TISSEEL, Baxter Deutschland GmbH, Unterschleißheim, Germany), and in addition secured with eight interrupted sutures (10.0 vicryl, Johnson & Johnson, Norderstedt, Germany) to the episclera. Next, a 2 mm wide strip of donor limbal tissue was dissected 360° from the corneoscleral button and was cut into small pieces and placed on the periphery and mid‐periphery of the amniotic membrane sparing the visual axis. Thereafter, fibrin glue (TISSEEL, Baxter Deutschland GmbH) was placed on the limbal pieces to attach them to the amniotic membrane. After 2–3 min, a bandage contact lens (Megasoft, Oculentis, Berlin, Germany) was placed on the ocular surface and left for 1 month. Thereafter, it was replaced by a new one for 2 more months.

### 2.3 Postoperative treatment

After surgery, preservative‐free 0.13% dexamethasone eye drops (Dexa EDO, Bausch & Lomb, Berlin, Germany) were administered 6 times daily for 4 weeks and then monthly reduced by one drop. Five months after surgery, patients remained on a frequency of 1–2 drops per day. Preservative‐free levofloxacin 0.5% eye drops (Oftaquix sine, Santen GmbH, Munich, Germany) were applied four times per day until the bandage contact lens was removed. Moreover, preservative‐free lubricating eye drops containing 3% trehalose and 0.15% hyaluronic acid (Thealoz® Duo, Thea Pharma GmbH, Berlin, Germany) were administered at least six times daily. Systemic therapy included mycophenolate mofetil (Cell‐Cept®, Roche Pharma AG, Grenzach, Deutschland) p.o. in all patients and prednisolone (Dekortin® H, Merck KGaA, Darmstadt, Germany) p.o. in some patients, applied at an initial dose of 1 mg/kg body weight and reduced within 1 month to 5 mg daily. After 6–8 weeks, systemic prednisolone administration was discontinued, and patients remained on systemic immunosuppression with mycophenolate mofetil alone.

### 2.4 Statistics

Statistical analysis was conducted by GraphPad Prism 6 (GraphPad Software Inc., La Jolla, CA, USA). AlloSLET graft survival probability was calculated by Kaplan–Meier survival analysis. For comparisons of Kaplan–Meier curves, the log‐rank (Mantel–Cox) test and the Gehan–Breslow–Wilcoxon test were used. Postoperative CDVA was compared for the first seven scheduled postoperative visits (48 months) with preoperative values, which resulted in seven comparisons, by a Wilcoxon matched pairs signed rank test. For the seven comparisons, the significance level α was adjusted by the Bonferroni correction to 0.007. No statistical comparisons were made at the 60th postoperative month, since this would include just four patients. Comparisons of recipient and donor/graft characteristics between patients, who developed epithelial defects suggestive of graft failure and patients that had a stable corneal epithelium were conducted by the Mann–Whitney *U*‐test. For these comparisons, the significance level was set to 0.05.

## 3 RESULTS

**FIGURE 1:**
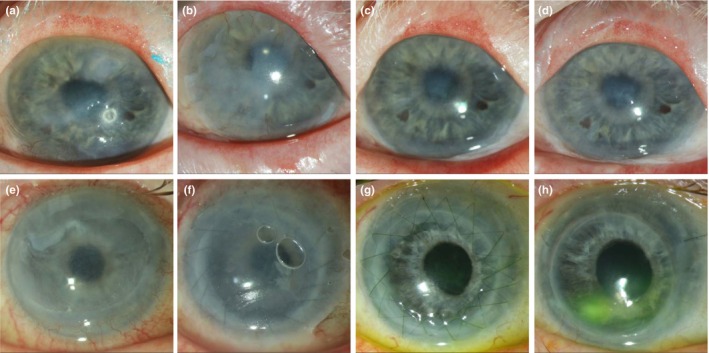
Slit‐lamp photographs of two eyes with total LSCD treated with alloSLET. In the upper row, the eye of a patient with ectrodactyly‐ectodermal dysplasia‐cleft (EEC) syndrome is shown. (a) Preoperative photograph showing conjunctivalization of the cornea. (b) One month post‐surgery, amniotic membrane remnants are visible under a bandage contact lens with superficial vascularization, which resolved after the bandage contact lens was removed. (c) Twelve months post‐surgery, a stable corneal epithelium with some subepithelial opacities is observed. (d) Thirty‐six months post‐surgery, the stable corneal epithelium persists with some subepithelial opacities. In the lower row, the eye of a patient with LSCD due to recurrent herpetic keratitis is presented. The eye had been treated by two amniotic membrane transplantations before we conducted alloSLET together with PK. (e) Preoperative photograph showing corneal ulceration. (f) One month post‐surgery, the cornea is still covered by the amniotic membrane, with several limbal pieces visible near the corneal centre and air bubbles between the amniotic membrane and the bandage contact lens. (g) Twelve months post‐surgery, the cornea is transparent, and the epithelium is closed, as indicated by negative fluorescein staining. (h) Thirty‐seven months post‐alloSLET with PK, the patient developed an inferior corneal epithelial defect (positive fluorescein staining) and anterior chamber flare, necessitating anterior chamber lavage. PCR of the aqueous humour sample was positive for herpes simplex virus 1, indicating herpetic keratitis. Informed consent was obtained from both patients for the publication of medical images.

Twenty‐four eyes of 20 patients (11 males and nine females), who received alloSLET because of persistent corneal epithelial defects due to total LSCD were identified. The mean patients' age was 58 ± 17 years (range 33–95). The median postoperative follow‐up period of all eyes was 36 months (range, 24–74 months). The surgery was uneventful in all cases. In Figure 1, the preoperative situation and postoperative pictures are presented for two patients. Twenty‐three (96%) of 24 eyes had an epithelialized corneal surface 1, 3 and 6 months after surgery, and 17 (71%) of 24 eyes displayed an epithelialized corneal surface 12 months after surgery.

**FIGURE 2:**
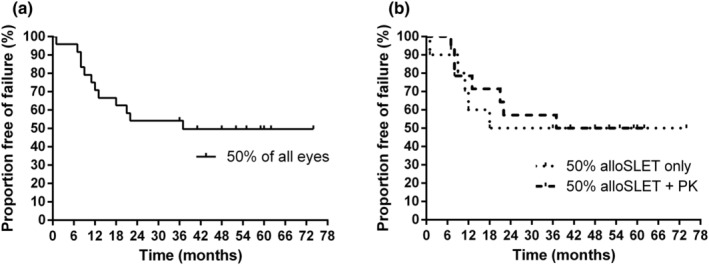
Kaplan–Meier analysis of stable corneal surface survival. (a) Survival analysis of all treated eyes (*n* = 24) revealed that 50% of the grafts survived until the last follow‐up visit. (b) Graft survival did not differ between alloSLET conducted alone or in combination with PK. Kaplan–Meier curves were statistically compared by the log‐rank (Mantel‐Cox) test and the Gehan‐Breslow‐Wilcoxon test. In eyes subjected to alloSLET alone 5 (50%) of 10 eyes had a stable corneal epithelium 36 months after surgery whereas in eyes treated by alloSLET with PK 8 (57%) of 14 eyes had a stable corneal epithelium after 36 months.

**FIGURE 3:**
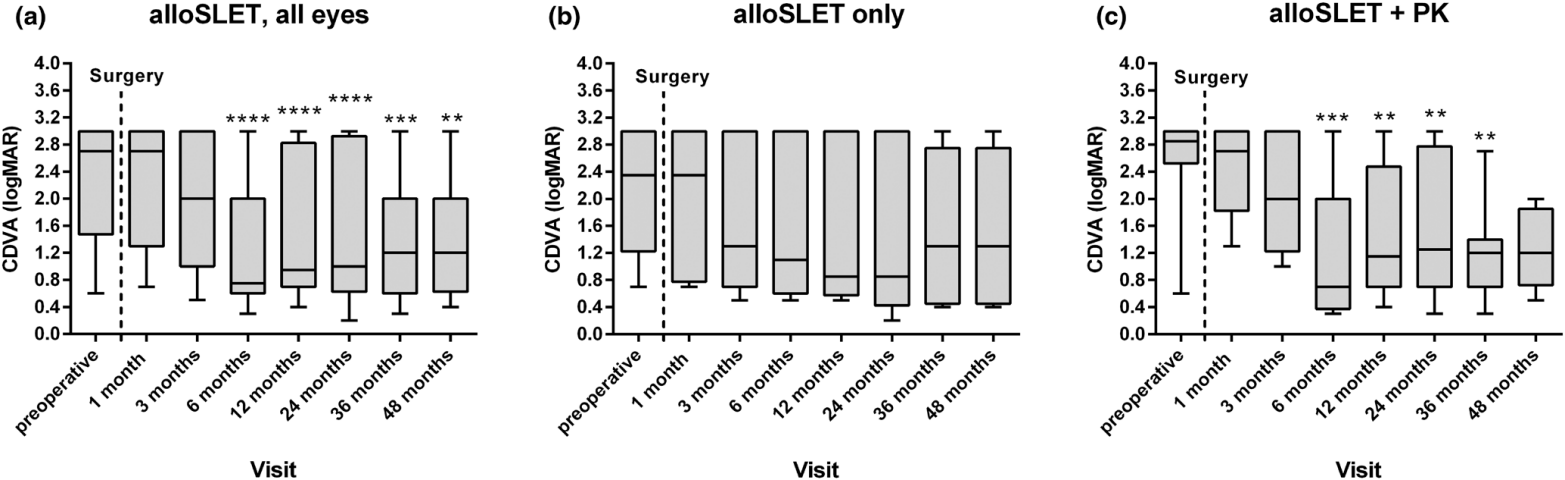
CDVA preoperatively and 1, 3, 6, 12, 24, 36 and 48 months after alloSLET. In the whole patient collective (*n* = 24, a), marked improvement of CDVA was observed after surgery. In eyes, which received alloSLET only (b) no significant improvement was observed, whereas in eyes that received alloSLET together with PK (c), marked improvement in CDVA was seen 6, 12, 24 and 36 months after surgery. Data are presented as box plots with minimum and maximum values. Postoperative CDVA values were compared with preoperative values by a Wilcoxon matched pairs signed rank test.

After 24 and 36 months, 13 (54%) of 24 eyes had a stable corneal surface with healthy corneal epithelium (Figure 2a). Survival of all alloSLET procedures, as determined by the maintenance of healthy corneal epithelium until the last follow‐up visit, was seen in 12 (50%) of 24 eyes. Remarkably, eyes that received alloSLET alone (*n* = 10) and alloSLET with PK (*n* = 14) did not differ in their graft failure rate (Figure 2b). For example, five (50%) of 10 eyes in the alloSLET only group and eight (57%) of 14 eyes in the alloSLET with PK group had a fully epithelialized corneal surface 24 and 36 months after surgery. In the whole patient collective, CDVA has markedly improved 6, 12, 24, 36 and 48 months following surgery compared to preoperative values (Figure 3a). However, in the subgroup of eyes that received alloSLET only, just a tendency towards improvement was seen (Figure 3b), whereas in the alloSLET with PK group a significant improvement was seen (Figure 3c). To assess whether certain characteristics of the recipients predisposed to graft failure, we compared the subgroup of eyes, in which the grafts survived with the subgroup in which the grafts failed with respect to intraocular pressure (IOP) elevation in the postoperative period, pre‐existing ocular diseases, recipients' age, gender and the number of previous amniotic and corneal grafts. Of the 24 eyes, 22 (92%) were pseudophakic. Notably, in the subgroup of eyes, in which the graft failed, as much as 10 (83%) of 12 experienced postoperative IOP elevation of which six (50%) had pre‐existing chronic glaucoma. Conversely, in the subgroup of eyes, in which the graft survived only five (42%) of 12 eyes experienced postoperative IOP elevation and two (17%) of them had pre‐existing chronic glaucoma (Tables 1 and 2). Moreover, we did not find an association between the reason of LSCD and the risk of graft failure because the underlying diseases were very variable (Tables 1 and 2). However, we noted that in the subgroup of eyes with failed limbal grafts seven (58%) of 12 had disease states associated with neurotrophic keratopathy, such as pervious radiotherapy, facial nerve palsy, herpetic keratitis or multiple corneal transplantations, whereas in the subgroup in which the grafts survived only 2 (17%) of 12 eyes had conditions associated with neurotrophic keratopathy (Tables 1 and 2).

**TABLE 1:** Characteristics of eyes, in which the graft survived until the last visit after surgery.

Patient	Age (years)	Gender (f/m)	Cause of LSCD	Other eye diseases	Elevated IOP	Systemic immunosuppression			Last visit after surgery (months)
						Starting	Ongoing	Duration (months)	
1	50s	M	Congenital aniridia	Pseudophakia	No	MMF 2 g/day	MMF 1 g/day	6	60
2, HLA	50s	M	Congenital aniridia	Pseudophakia	No	MMF 2 g/day	MMF 1 g/day	6	48
3	70s	M	Congenital aniridia	Pseudophakia	No	MMF 2 g/day	MMF 1 g/day	12	36
4*	40s	F	Chemical burn	Pseudophakia	Yes	MMF 2 g/day, prednisolone 1 g/kg BW	MMF 2 g/day	14	62
5*	70s	M	Chemical burn	Pseudophakia, glaucoma	Yes	MMF 2 g/day, prednisolone 1 g/kg BW	MMF 2 g/day	15	55
6*	30s	F	Unknown	None	No	MMF 2 g/day	MMF 2 g/day	16	52
7*	30s	F	Unknown	None	No	MMF 2 g/day	MMF 2 g/day	12	48
8*	40s	F	Several corneal transplantations due to juvenile glaucoma	Pseudophakia, glaucoma	Yes	MMF 2 g/day, prednisolone 1 g/kg BW	MMF 2 g/day	13	60
9*	40s	F	Acanthamoeba keratitis	Pseudophakia	Yes	MMF 0.5 g/day	MMF 0.5 g/day	3	59
10*	70s	M	Sjörgen's syndrome	Pseudophakia, epiretinal gliosis	Yes	MMF 2 g/day, prednisolone 1 g/kg BW	MMF 2 g/day	12	41
11	50s	F	EEC syndrome	Pseudophakia	No	MMF 2 g/day, prednisolone 1 g/kg BW	MMF 2 g/day	32	74
12	80s	F	Several corneal transplantations after bacterial keratitis	Pseudophakia	No	MMF 2 g/day	MMF 2 g/day	12	37

*Note*: Age (years) at surgery; gender: female (f), male (m); cause of limbal stem cell deficiency (LSCD); other eye diseases, postoperatively elevated IOP, systemic immunosuppression (starting daily dose, ongoing daily dose duration) and last visit of the patient after surgery.

* indicates that alloSLET was combined with penetrating keratoplasty.

Abbreviations: AMD, age‐related macular degeneration; BW, body weight; EEC syndrome, ectrodactyly‐ectodermal dysplasia‐cleft syndrome; LSCD, limbal stem cell deficiency; MMF, mycophenolate mofetil.

**TABLE 2:** Characteristics of eyes experiencing graft failure.

Patient	Age (years)	Gender (f/m)	Cause of LSCD	Other eye diseases	Elevated IOP	Systemic immunosuppression			Failure after surgery	
						Starting	Ongoing	Duration (months)	Time (months)	Reason
1	50s	M	Chemical burn	Pseudophakia, glaucoma	Yes	MMF 2 g/day, prednisolone 1 g/kg BW	MMF 2 g/day	12	11	Corneal erosion
2	40s	M	Chemical burn	Pseudophakia, glaucoma	Yes	MMF 2 g/day, prednisolone 1 g/kg BW	MMF 2 g/day	9	9	Corneal erosion
3*	40s	M	Blast injury	Pseudophakia, glaucoma	Yes	MMF 2 g/day, prednisolone 1 g/kg BW	MMF 2 g/day	18	8	Conjunctivalization
4	40s	M	Blast injury	Pseudophakia, glaucoma	Yes	MMF 2 g/day, prednisolone 1 g/kg BW	MMF 2 g/day	18	18	Corneal ulcer
5	60s	M	Graft versus host disease	Pseudophakia, glaucoma	Yes	MMF 1 g/day	MMF 1 g/day	3	1	Corneal erosion
6*	50s	M	Several corneal transplantations due to keratoconus	Pseudophakia, uveitis	Yes	MMF 2 g/day, prednisolone 1 g/kg BW	MMF 2 g/day	12	13	Graft rejection
7*, HLA	60s	M	Radiotherapy due to lymphoid hyperplasia of the orbit	Pseudophakia	Yes	MMF 2 g/day, prednisolone 1 g/kg BW	MMF 2 g/day	7	7	Bacterial keratitis
8*, HLA	50s	W	Facial nerve palsy with neurotrophic keratopathy and chronic exposition keratopathy	Pseudophakia	No	MMF 2 g/day, prednisolone 1 g/kg BW	MMF 2 g/day	13	21	Bacterial keratitis
9*	80s	M	Herpetic keratitis/bacterial keratitis/corneal transplantation	Pseudophakia, AMD	Yes	MMF 2 g/day, prednisolone 1 g/kg BW	MMF 2 g/day	13	37	HSV‐1 keratitis
10*	40s	W	Several corneal transplantations due to juvenile glaucoma	Pseudophakia, glaucoma	Yes	MMF 2 g/day, prednisolone 1 g/kg BW	MMF 2 g/day	12	22	Graft rejection
11*	40s	W	Several corneal transplantations due to keratoconus	Pseudophakia	Yes	MMF 2 g/day, prednisolone 1 g/kg BW	MMF 2 g/day	8	8	CMV keratitis
12	90s	M	Exposition keratopathy, neurotrophic keratopathy	Pseudophakia, AMD	No	MMF 2 g/day	MMF 2 g/day	12	12	Bacterial keratitis

*Note*: Age (years); gender: female (f), male (m); diagnosis; number of previous amniotic membrane (AM) transplantations; number of previous perforating keratoplasty (PK).

* Stars indicates that alloSLET was combined with penetrating keratoplasty (PK). ‘HLA’ indicates that the graft was HLA‐matched.

Abbreviations: AMD, age‐related macular degeneration; CMV, cytomegalovirus; HSV‐1, herpes simplex virus type 1; LSCD, limbal stem cell deficiency; MMF, mycophenolate mofetil.

**FIGURE 4:**
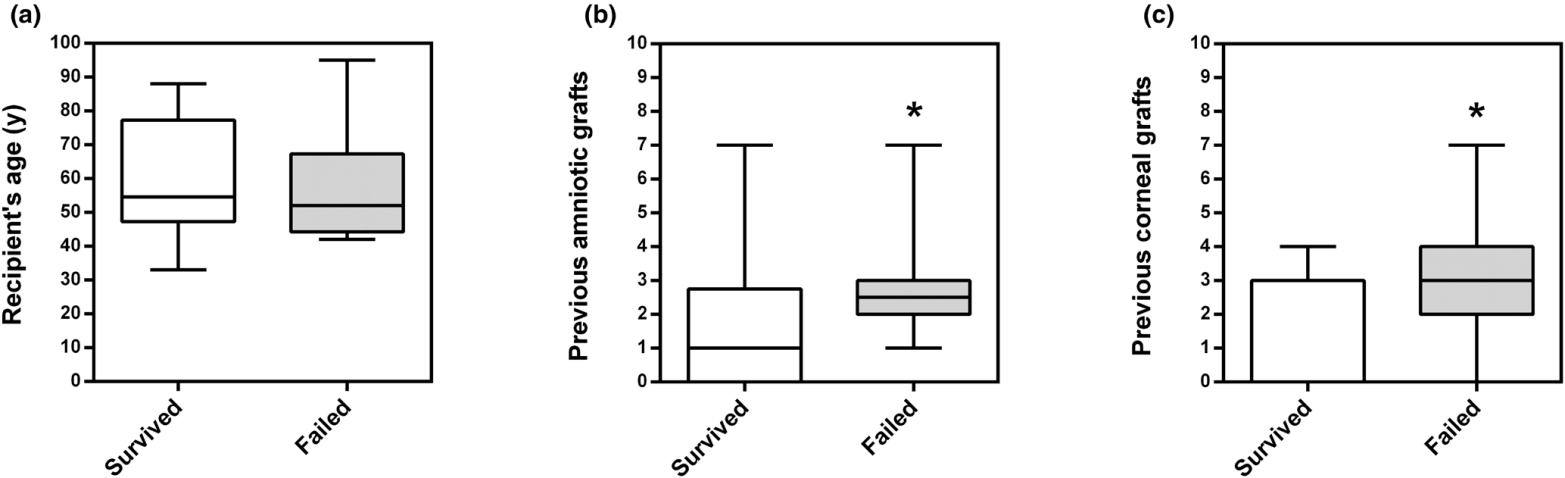
Potential risk factors of recipients that may have increased the risk of graft failure. (a) Recipients' age was similar in the group, in which the graft survived compared the one, in which the graft failed. (b) Remarkably, the group of eyes in which the graft failed had a markedly higher number of previous amniotic membrane transplantations. (c) Likewise, the number of previous corneal grafts was higher in eyes, in which the graft failed (median: three previous grafts) compared to eyes in which the graft survived (median: 0 previous grafts) (*n* = 12 per group). Data are presented as box plots with minimum and maximum values. The Mann–Whitney *U*‐test was used for statistical comparison of the groups.

Recipients' age at the time of transplantation was similar in eyes, in which the alloSLET graft survived and those experiencing graft failure (Figure 4a). Notably, in the subgroup of eyes experiencing graft failure nine (75%) of 12 were males and only three (25%) of 12 females, whereas in the subgroup without graft failure the gender distribution was more balanced with five (42%) of 12 eyes being males and seven (58%) of 12 females (Tables 1 and 2).

Remarkably, in the subgroup of eyes experiencing graft failure, the number of previous amniotic membrane transplantations (Figure 4b) and corneal transplantations (Figure 4c) was higher than in the subgroup, in which the graft survived.

**FIGURE 5:**
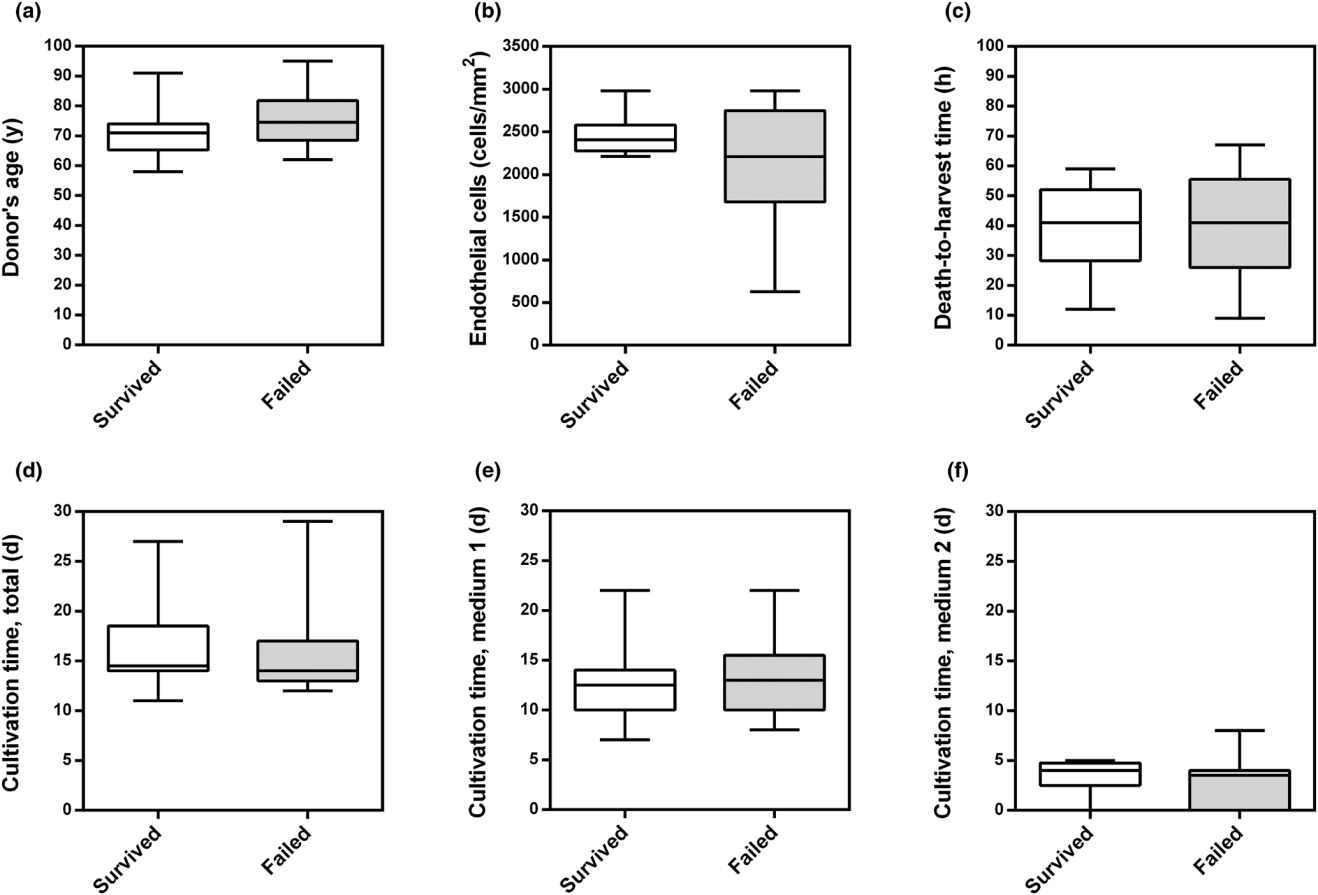
Potential risk factors of donor tissue characteristics or harvesting/culturing conditions that may have reduced the quality of the transplanted graft pieces. (a) Donors' age (years, y), (b) endothelial cell density (cells/mm^2^), (c) time between the donors' death and harvesting of the graft tissue in hours (h), (d) total time of graft tissue cultivation in days (d), (e) cultivation time in medium 1 (Cat. No. F‐9016; Biochrom, Berlin, Germany) and (f) cultivation time in medium 2 (Cat. No. F‐9017; Biochrom) are presented for the grafts that survived (*n* = 12) and for those that failed (*n* = 12). In both media, corneoscleral buttons were stored at 34°C. Data are presented as box plots with minimum and maximum values. The Mann–Whitney *U*‐test was used for statistical comparison of the groups.

To investigate whether certain donor/graft characteristics or harvesting/culturing conditions were a risk factor for graft failure, we compared the subgroups of eyes, in which the grafts survived and failed with regard to donor's age, endothelial cell density, death‐to‐harvest time, total cultivation time and cultivation time in medium 1 and medium 2. Of note, none of these factors seemed to have an influence on graft failure (Figure 5). In the present study, only three grafts serving for alloSLET have been human leukocyte antigen (HLA)‐matched, and two of them failed. Because of the low number of HLA‐matched grafts, no conclusion with regard to their advantage over non‐matched grafts can be drawn from this study. With respect to postoperative ocular side effects, elevated IOP was the most common. As many as 15 (63%) of 24 eyes experienced periods of IOP elevation in the postoperative period although only eight (33%) of 24 eyes had pre‐existing glaucoma, indicative of steroid responsiveness as a contributing factor. In six (25%) of 24 eyes, pieces of limbal tissue dislocated to the centre of the cornea apparently affecting vision. Although the pieces typically resolved within 6 months, in some cases remaining limbal pieces were observed even after 24 months. One (4%) of the 24 eyes frequently lost its bandage contact lens resulting in graft failure already 1 month after transplantation.

## 4 DISCUSSION

This study presents the outcomes of patients monitored for a minimum of 2 years following the treatment of non‐healing corneal epithelial defects using alloSLET for which cultured corneoscleral buttons from deceased donors were used. The subjects suffered from total LSCD of various origins and were ineligible for autologous limbal tissue treatment. One year post‐surgery, 71% of treated eyes exhibited a stable corneal epithelium, which decreased to 54% after 2 and 3 years after surgery, respectively.

Of note, limited literature addresses mid‐ or long‐term results after alloSLET. For example, a case report on bilateral alloSLET in combination with PK described a stable epithelium after 2 years in one patient with chemical injury (Kunapuli & Fernandes, 2021). A study with 18 eyes of 17 patients who underwent alloSLET in the early stage of ocular chemical injury evaluated the mean time to epithelialization, which was 22.5 ± 9.14 days (Iyer et al., 2017). Notably, 94.11% of the eyes achieved complete corneal epithelialization in the immediate postoperative period (Iyer et al., 2017), which is similar to our previous and present findings (Riedl et al., 2021). Furthermore, Agarwal et al. (2020) compared the outcomes of alloSLET versus amniotic membrane grafting in early management of severe grade ocular chemical injury and reported on a faster epithelial healing in the alloSLET group. AlloSLET also reduced the need for subsequent surgeries compared to amniotic membrane transplantation (Agarwal et al., 2020). Recently, Shanbhag et al. reported their experience with alloSLET in chronic bilateral LSCD in 30 eyes of 29 patients. Sixteen eyes of 16 patients who underwent live‐related alloSLET without HLA or ABO matching were compared with 14 eyes of 13 patients who underwent cadaveric alloSLET with the same postoperative pulse intravenous immunosuppression regimen using methylprednisolone (Shanbhag et al., 2019). Successful outcomes were reported in 14 (87.5%) of 16 eyes in the live‐related donor group and in 11 (78.6%) of 14 eyes in the cadaveric donor group 1 year postoperatively and at the final follow‐up (median 28 months, range 13–66 months) (Shanbhag et al., 2019). Our 1‐year outcome of 71% is comparable with that in the cadaveric donor group reported by Shanbhag et al. (2019) 1 year after surgery. However, while Shanbhag et al. reported no further failures after the first postsurgical year, our success rate dropped from 71% 1 year postoperatively to 54% 2 and 3 years after surgery, and to 50% thereafter.

Several reasons may account for these differences. One reason might be the postoperative systemic immunosuppressive treatment, which differs between the present study and the study of Shanbhag et al. While we applied mycophenolate mofetil directly after surgery orally, Shanbhag et al. (2019) applied a pulse intravenous immunosuppression regimen starting with 500 mg of methylprednisolone. The advantage of the pulse intravenous immunosuppression regimen, which involves the intermittent administration of steroids at supra‐pharmacological doses, is that it does not rely on patient compliance and is believed to be cumulatively less toxic than continuous steroid treatment at lower doses. However, administering steroids intravenously presents logistical challenges and is associated with an increased risk of various side effects, including hemodynamic abnormalities, seizures, abnormal behaviour, hyperglycaemia, hypokalaemia and infections (Sinha & Bagga, 2008).

So far, no study has been reported that directly compared both immunosuppressive schemes with regard to limbal graft rejection. However, another study that utilized living‐related allogeneic donor tissue for SLET and mycophenolate mofetil postoperatively, reported on similar outcomes as Shanbhag et al., suggesting that both immunosuppressive schemes may be similarly effective (Prabhasawat et al., 2021).

Notably, in the present study, the immunosuppressive regimen was similar in the group of eyes, in which the grafts survived, as in the group experiencing graft failure. Moreover, just two of the failed grafts had clear signs of graft rejection, whereas the other nine failures were due to keratitis, conjunctivalization or corneal erosion, which may either be a result of chronic subtle rejection or of stem cell depletion due to unfavourable conditions of the stem cell niche.

Remarkably, a recent study employing genetic analysis of corneal epithelial cells on patients receiving alloSLET for total LSCD from live‐related donors reported that donor cells were detectable on the corneal surface for up to 30 months after transplantation (Chotikavanich et al., 2023). However, absence of donor cells did not mean that the epithelial transplants failed. Interestingly, in most patients with successful outcomes, the recipient genotype was found. Based on these findings, it has been suggested that the donor grafts themselves may not be the only limbal stem cell source in the repopulation of the corneal epithelial cells but may have other roles, especially in conjunction with the properties of the transplanted amniotic membrane in providing a niche for the patients' own remaining stem cells to repopulate or for conjunctival epithelial cells to differentiate into a corneal epithelial phenotype (Chotikavanich et al., 2023). Taken together, the mechanism that maintains the long‐term clinical successful outcome without evidence of viable stem cells remains to be elucidated. Given that 2–3 years after alloSLET, no donor cells are detected on the corneal surface, the question arises how long and to which extent immunosuppression is needed.

Other differences between the study of Shanbhag et al. and the present study may be the criteria for graft selection and patients' characteristics. For example, Shanbhag et al. suggested that harvesting tissue from living donors seems to be preferable, because limbal cells obtained from cadaveric donor eyes were reported to have a reduced proliferation rate in vitro and a blunted epithelialization rate in vivo (Shanbhag et al., 2019; Shimazaki et al., 2007; Vemuganti et al., 2004). This suggestion is supported by findings of Prabhasawat et al. (2021), who reported on a survival rate of 73.2% after 3 years in eyes that received living‐related allogeneic SLET for LSCD.

We agree that using fresh tissue may be advantageous with regard to graft survival. However, the availability of living‐related donors is limited compared to cadaveric donor eyes. Moreover, utilizing tissue from deceased‐donor eyes increases the flexibility in planning the surgery. With regard to the use of cadaveric donor eyes for alloSLET, Shanbhag et al. (2019) suggested to use tissue from patients <60 years and fresh tissue, preferably before 48 h after harvesting. However, in many eye banks, a time interval of less than 48 h is not feasible due to logistical issues, such as the waiting time for microbiological or serological results. Moreover, young donors are hardly available. In our study, we utilized limbal graft tissue with a time in culture from 11 to 29 days. Also, the donors' age was relatively high in the present study ranging from 58 to 95 years. Remarkably, our results do not suggest that grafts from older donors or after a longer cultivation time have a worse survival rate. However, we agree that this does not rule out the possibility that freshly harvested donor tissue or tissue from very young donors might have a better survival probability.

Notably, in the subgroup of eyes experiencing graft failure, 75% were males and only 25% were females. In contrast, in the subgroup without graft failure, 42% of the eyes were from male patients and 58% were from female patients. The reason for this difference is difficult to determine from our data, but one possible explanation could be differences in adherence to postoperative therapy.

Intriguingly, in the subgroup of eyes that experienced graft failure in the present study, 83% had postoperative IOP elevation and 50% had pre‐existing glaucoma. In contrast, in the subgroup of eyes, in which the graft survived, only 42% experienced postoperative IOP elevation and only 17% had chronic glaucoma. Moreover, the eyes that experienced graft failure had also markedly more previous amniotic membrane and corneal graft transplantations. These findings suggest that elevated IOP and a history of amniotic membrane or corneal transplantations may be risk factors for graft failure. Elevated IOP, along with the medication required for its treatment, and previous corneal surgeries might contribute to a more pronounced immune response, counteracting graft survival. Notably, we also observed a higher incidence of conditions associated with neurotrophic keratopathy, such as facial nerve palsy, herpetic keratitis and repeated corneal transplantations, in the group with graft failure compared to the group with successful grafts. This indicates that neurotrophic keratopathy may be linked to a higher rate of alloSLET failure. Growth factors released from corneal nerve endings may be crucial for the survival of limbal stem cells, suggesting that re‐innervation strategies should be considered before limbal stem cell transplantation.

Remarkably, we did not find differences in stem cell graft failure between eyes that received alloSLET alone and those that received alloSLET combined with PK. The combined procedure was performed in eyes with deep corneal ulcers or stromal scars. While some surgeons prefer to stabilize the surface first with alloSLET alone, followed by a secondary PK, we chose the combined procedure for several reasons. First, in some of our patients, the cornea was very thin, posing a risk of perforation, which influenced our decision to opt for the combined approach. Second, in transplantation techniques utilizing small limbal fragments, including alloSLET, the stem cells are thought to be distributed across the corneal surface during the postoperative phase. Histopathological and ultra‐high‐resolution optical coherence tomography studies demonstrated persistence of the human amniotic membrane while epithelial cells stratified over it (Amescua et al., 2014; Basu et al., 2016). Since the amniotic membrane plays a critical role in promoting and preserving the stemness of limbal epithelial stem cells (Chen et al., 2015), its persistence after SLET may contribute to the prolonged success of the procedure. Even many months after SLET, both epithelial progenitor cells and limbal epithelial stem cells have been detected in the basal layers of the regenerated epithelium next to the retained amniotic membrane (Basu et al., 2016). It is unclear whether these cells later migrate to the corneal periphery or the limbal niche. Given that in cases of total LSCD, the niche is damaged, it is likely that the transplanted stem cells remain dispersed over the corneal surface. This supports the argument for an all‐in‐one approach rather than a fractionated one, where removing the central portion of the cornea, including the epithelium with stem cells, would result in their depletion. Another disadvantage of performing alloSLET followed by secondary PK is the requirement for two surgeries and the use of two donor tissues.

Notably, some previous studies suggested that a combined procedure of autologous SLET and keratoplasty may be associated with a higher rate of stem cell transplant failure (Basu et al., 2016; Gupta et al., 2018; Vazirani et al., 2016). However, the absolute and relative numbers of eyes treated with the combined procedure were quite small in these studies. Moreover, the need for simultaneous keratoplasty may suggest a greater severity of the original trauma leading to LSCD, which and may have contributed to the higher failure rate in these studies. Hence, the risk factor of combined surgery for stem cell transplant failure should be interpreted with caution and is not supported by the present study.

To unveil whether some donor or tissue processing characteristics had an influence on the surgical outcome, we compared donors' age, endothelial cell density, death‐to‐harvest time and the time in culture between the eyes that experienced graft failure and those, in which the graft survived. Remarkably, none of these factors differed significantly between the grafts that survived and those that failed. However, we observed that none of the grafts that had an endothelial cell density below 2000 cells/mm^2^ survived. This might be a hint that the limbal stem cell quality is reduced in grafts with a very low endothelial cell density.

Of note, our reported outcome of alloSLET is comparable to the results reported by other groups applying CLET with allogeneic tissue expander on intact amniotic membrane, which reported a survival rate of 42%–50% after more than 2 years (Pauklin et al., 2010; Shimazaki et al., 2007). The survival rate of most studies using non‐HLA‐matched KLAL was also reported in a similar range after at least 2 years following transplantation (Cheung et al., 2020; Ilari & Daya, 2002; Miri et al., 2010; Ozer et al., 2020). However, compared to allogeneic CLET, alloSLET has the advantage of being cheaper and available with less logistical and technical effort. Moreover, alloSLET has the advantage over KLAL of being easier to perform and potentially reversible.

Since, unlike KLAL, the limbal pieces transplanted during alloSLET do not come into close contact with the recipient's limbus, the procedure may be less immunogenic than KLAL.

HLA‐matched grafts were no exclusion criterion when we planned the study, since we expected to gain useful information regarding the survival rate of HLA‐matched and non‐matched grafts. So far, the evidence for HLA matching in corneal graft rejection prophylaxis is controversial (Böhringer et al., 2018; Jiang et al., 2022). Unfortunately, we identified just three HLA‐matched grafts among our patients, which is not enough to qualify for statistical analysis. Interestingly, two of three eyes that received HLA‐matched grafts experienced graft‐failure. However, due to the limited number of eyes, we cannot draw conclusions from these findings.

The advantage of this study is its relatively long follow‐up time, and the standardized surgical procedure, postoperative treatment, and patient visits. However, the retrospective study design has its weaknesses. For example, no collection of corneal tissue for further scientific analysis was done in this study. Additionally, the patient collective was heterogeneous, making it difficult to draw conclusions regarding the treatment success of an individual aetiology of LSCD.

In summary, ocular surface reconstruction in patients with LSCD remains a challenging procedure, especially when both eyes are affected. The primary outcome of the study was an epithelialized corneal surface with healthy corneal epithelium, observed in 71% of the eyes 12 months after surgery, 54% at 24 and 36 months postoperatively, and 50% thereafter. These results are comparable to other allogeneic transplantation techniques, such as allogeneic CLET or KLAL. The advantage of alloSLET is that it is relatively easy to perform and requires less financial, logistic and technical effort than allogeneic CLET and is less invasive than allogeneic KLAL. Therefore, alloSLET can be more easily repeated in case of graft failure compared to the other two techniques, making it a potentially good choice for patients without available autologous donor tissue. Our findings indicate that male gender, neurotrophic keratopathy, multiple previous corneal surgeries and elevated IOP may increase the risk of alloSLET failure. Hence, IOP should be regularly monitored in these patients.

## AUTHORS CONTRIBUTIONS

Conceptualization, J.C.R. and A.G.; Methodology, J.C.R.; Software, A.G.; Validation, J.C.R. and A.G.; Formal Analysis, J.C.R. and A.G.; Investigation, J.C.R., B.C.G., A.M., J.W. and A.G.; Resources, J.C.R., A.G., and N.P.; Data Curation, J.C.R., A.M., B.C.G. and A.G.; Writing – Original Draft Preparation, J.C.R. and A.G.; Writing – Review and Editing, J.C.R., B.C.G., J.W., F.B. and A.G.; Visualization, J.C.R., B.C.G.; Supervision, A.G.; Project Administration, J.C.R., B.C.G., J.W. and A.G.

## CONFLICT OF INTEREST STATEMENT

J.C.R., J.W.P., B.C.G., F.B., N.P., A.M. and A.G. state that there is no conflict of interest. It is assured that there are no links with a company whose product is mentioned in the article or with a company that sells a competitive product. The presentation of the topic is independent and the presentation of the content is product‐neutral.

## ETHICS STATEMENT

According to local law (‘Landeskrankenhausgesetz’ §36, §37), no ethical approval was required for this retrospective analysis.
